# Assessing the Relationship Between the Psychosocial Impact of Dental Aesthetics, Self-Esteem, and Dental Habits

**DOI:** 10.3390/dj14030165

**Published:** 2026-03-12

**Authors:** Mara Ștefania Șimon, Alexandru Grațian Grecu, Ioana Maria Colceriu-Șimon, Andrea Maria Chisnoiu, Cristina Gasparik, Diana Dudea

**Affiliations:** 1 Department of Prosthetic Dentistry and Dental Materials, Iuliu Hațieganu University of Medicine and Pharmacy, 400012 Cluj-Napoca, Romania; simon_mara_stefania@elearn.umfcluj.ro (M.Ș.Ș.); maria.chisnoiu@umfcluj.ro (A.M.C.); gasparik.cristina@umfcluj.ro (C.G.); ddudea@umfcluj.ro (D.D.); 2 Department of Orthodontics, Iuliu Hațieganu University of Medicine and Pharmacy, 400012 Cluj-Napoca, Romania; simon.ioana@umfcluj.ro

**Keywords:** self-esteem, self-perception, oral health behaviour, psychosocial impact of dental aesthetics questionnaire, rosenberg self-esteem, quality of life

## Abstract

**Background/Objectives**: Dental aesthetics has been shown to be associated with psychosocial functioning and self-perception, underscoring the importance of integrating psychosocial considerations into holistic patient care. This study assessed self-perceived dental aesthetics, self-esteem, and dental habits among dental medicine students in relation to socio-demographic factors and dental knowledge. **Methods**: A cross-sectional survey of 172 students from the Dental Medicine Faculty in Cluj-Napoca, Romania, was conducted. The sample was divided into two groups, based on their prior education in dentistry: Level 1 students at the beginning of their studies, without any prior dental medicine experience, and Level 2 students with basic knowledge in the field of dentistry at the end of their preclinical years. After informed consent, participants completed the Psychosocial Impact of Dental Aesthetics Questionnaire (PIDAQ), the Rosenberg Self-Esteem Scale (RSES), and questions on oral hygiene and socio-demographics. Subscale and overall questionnaire scores were computed and statistically analysed using Pearson’s correlations and independent-samples *t*-tests to examine relationships among self-perceived dental aesthetics and self-esteem and differences between variables. **Results**: A negative correlation was found between the psychosocial impact of dental aesthetics and overall self-esteem scores (*p* = 0.006). Greater aesthetic concerns were associated with lower self-esteem (*p* = 0.003), although the magnitude of correlation was small. Female participants had higher social impact scores for dental aesthetics (*p* = 0.039), whereas male students reported higher self-esteem (*p* = 0.047). Students from Level 2 group presented higher self-esteem than Level 1 (*p* = 0.016). Regarding oral hygiene, a small but statistically significant association was found between dental flossing frequency and aesthetic concern scores (*p* = 0.044). Additionally, individuals who frequently attended dental check-ups reported a more favourable self-image. **Conclusions**: Self-perceived dental aesthetics correlates with self-esteem among dental students, varying by gender and academic level. The Dental Self-Confidence subscale of the PIDAQ had the greatest perceived impact, underscoring the importance of self-image for dental students’ psychological and social well-being. Dental flossing and regular dental consultations appear to be linked to improved self-perceived dental aesthetics.

## 1. Introduction

Since ancient times, humans have been social beings, and the need for acceptance within communities has always been crucial for survival and well-being. During social interactions, the orofacial region plays an essential role in conveying words, emotions, and mental or physical states [1].

In today’s society, where adherence to aesthetic norms is highly valued, deviations from beauty standards can significantly affect mental health and quality of life, especially among younger generations [2]. Studies show that minor imperfections in dental aesthetics can lead to social withdrawal, low self-confidence, negative comparisons with others, and even more serious mental health issues such as anxiety and depression [3,4,5,6].

At the other end of the spectrum, high self-esteem—also influenced by well-perceived dento-facial aesthetics—promotes a better quality of life through enhanced social interactions and more effective management of life events. These factors also play a critical role in guiding clinical protocols during the planning phase of complex dental aesthetic treatments [7,8,9].

Studies indicate that an individual’s self-image regarding dento-facial appearance can also be influenced by their dental hygiene routines. Good oral hygiene habits, including regular tooth brushing, use of mouthwash or flossing, and periodic visits to the dental office for prophylactic check-ups, can reduce concerns about dental aesthetics by lowering the risk of cavities or periodontal disease [10,11].

The Psychosocial Impact of Dental Aesthetics Questionnaire (PIDAQ), developed by Klages et al. [12], is an objective assessment tool that measures the self-perceived impact of dental aesthetics on an individual’s psychological and social aspects. Comprising 23 items organised into four main domains—Dental Self-Confidence (DSC), Social Impact (SI), Psychological Impact (PI), and Aesthetic Concern (AC)—the questionnaire assesses personal perceptions, social fears, feelings of inferiority, and aesthetic concerns related to dental appearance [12]. It has been widely translated and validated across many populations, demonstrating its broad relevance and enabling a comprehensive understanding of how dental aesthetics influence social life and overall well-being [13,14,15].

Studies have shown that individuals with high levels of aesthetic concerns tend to have lower self-confidence and experience greater psychological and social distress, underscoring the direct impact of self-perception on dental treatment outcomes, including orthodontic or prosthodontic procedures [16,17,18].

Complementary to this, the Rosenberg Self-Esteem Scale (RSES), developed in 1965, is a widely used unidimensional measure of self-esteem. It comprises 10 questions, with half addressing positive aspects and the other half addressing negative aspects, thereby providing insight into an individual’s overall sense of self-worth [19]. The link between self-esteem and dental factors is important because self-esteem can significantly influence how people perceive their dental treatment outcomes and their overall satisfaction with their appearance [20,21]. Successful dental treatments not only improve aesthetic outcomes but also significantly enhance patients’ self-esteem and overall quality of life [22,23].

Psychosocial assessment tools have become even more relevant in the context of the COVID-19 pandemic. As observed by Alqarawi et al., global crises can amplify aesthetic-related anxieties during difficult times, underscoring the importance of dento-facial appearance on the psychological and social health of individuals [24]. Even the transition back to normal social interactions without safety masks or distancing may have further intensified these concerns [25,26]. This present study’s data collection was conducted in a post-pandemic period, providing a contemporary look at psychosocial outcomes during the transition back to mask-free social and clinical interactions.

While most studies rely on the use of PIDAQ related to clinical malocclusion, research investigating psychosocial impact (PIDAQ) with general self-esteem (RSES), alongside the additional variables of oral hygiene habits and levels of education, remains overlooked in the literature [4,5,6,8,9,13,14,15,16,17,18,21]. Studying these psychosocial dynamics is essential for future dental professionals, as their self-perception may shape their professional approach regarding holistic patient care.

Additionally, a significant gap in scientific literature exists regarding the pre-clinical dental students in Eastern Europe. The dental student population has a unique double role as ‘professional aesthetic observer’ and future aesthetic treatment providers. Their specific training likely improves their awareness of alterations from standards regarding dento-facial aesthetic parameters. Addressing this gap may contribute to a better understanding of how early professional education interacts with self-perception during formative academic stages.

Therefore, this study assesses self-perceived dental aesthetics, self-esteem, and dental habits among dental students in relation to socio-demographic factors.

Based on the study’s purpose, the following exploratory research questions were developed:

1. Are there any significant correlations between self-perceived dental aesthetics (as measured with PIDAQ) and self-esteem (as measured with RSES)?
2. Are there any significant differences in self-perceived dental aesthetics, self-esteem and dental hygiene habits, with respect to the variables gender and dental medical knowledge?

## 2. Materials and Methods

Prior to the study, ethical approval was obtained from the Research Ethics Committee of “Iuliu Hațieganu” University of Medicine and Pharmacy in Cluj-Napoca, under approval number DEP245/30.10.2024 from 30 October 2024. The study was conducted in accordance with the Declaration of Helsinki. All study participants signed an informed consent form approved by the Research Ethics Committee of “Iuliu Hațieganu” University of Medicine and Pharmacy in Cluj-Napoca. This study was funded by the Research Project PCD Nr. 284/51/12.01.2026 from “Iuliu Hațieganu” University of Medicine and Pharmacy in Cluj-Napoca. This article was also supported by project no. 100418/29.08.2025, SMIS code 350525, financed by the Ministry of Investments and European Projects, through the Health Programme. The research used the infrastructure provided by the University of Medicine and Pharmacy “Iuliu Hatieganu”-Cluj-Napoca, Romania.

A cross-sectional survey was conducted via a convenience sampling method from a single academic centre, with a final sample of 172 students enrolled in the Faculty of Dental Medicine at “Iuliu Hațieganu” University of Medicine and Pharmacy in Cluj-Napoca, Romania. Informed consent and GDPR compliance were obtained from all participants before their participation. A priori power analysis using G*Power (v3.1) estimated the sample size based on the following parameters: effect size d of 0.5; α error probability of 0.05; power of 0.8; and an allocation ratio of 1 [27]. Based on these inputs, the minimum sample size was 128.

The inclusion criteria were enrollment as an undergraduate dental student, age over 18 years, ability to understand the information given in the study questionnaire, and provision of written informed consent.

Exclusion criteria included refusal to participate, incomplete questionnaires, missing responses on key outcome variables (PIDAQ or RSES total scores), or withdrawal of consent. Questionnaires with substantial missing or inconsistent data were excluded from the final statistical analysis.

In the present study, the categorization into “Level 1” and “Level 2” does not reflect pre-clinical versus clinical training. Level 1 refers to newly enrolled dental students at the beginning of their preclinical studies, who are primarily engaged in foundational biomedical sciences with minimal exposure to dental-specific theoretical content. Level 2 refers to dental students at the end of their preclinical phase, who have increased exposure to introductory dental subjects and simulation-based pre-clinical training, but still no independent clinical patient management.

The following questionnaires were administered to the study sample: the Psychosocial Impact of Dental Aesthetics Questionnaire (PIDAQ) [12], the Rosenberg Self-Esteem Scale (RSES) [19], and an additional questionnaire comprising items on dental habits, including how often participants brush their teeth daily, whether they use dental floss or mouthwash, and the frequency of visits to the dental office in the last five years. The extended version of this questionnaire, including the GDPR form and informed consent form, is available in the Supplementary file under the name: “Questionnaire S1: Assessing The Relationship between the Psychosocial Impact of Dental Aesthetics, Self-Esteem, and Dental Habits”.

The questionnaires were administered in a self-completion format and took approximately 20 min to complete. From the total of 176 initial responses, four questionnaires were excluded due to incomplete answers after a manual review for data integrity. Consequently, a final sample of 172 participants was included in the statistical analysis, with a response rate of 97.7%. The collected data were recorded in Microsoft Excel spreadsheets. A comparative statistical analysis of the data was conducted across various monitored parameters, using relevant statistical tests and descriptive analyses. Subscale and overall questionnaire scores were calculated and analysed, and then used in descriptive and inferential analyses (Pearson correlations and *t*-tests) to examine the relationship between self-perceived dental aesthetics and self-esteem, as well as differences in these constructs by gender and stage of dental education. In line with current protocols involving psychometric research, the total scores for PIDAQ and RSES were treated as continuous variables, as sum scores from multi-item scales typically approximate an interval level of measurement [28,29]. The effect size was also calculated.

## 3. Results

The final sample consisted of 172 students from the Faculty of Dental Medicine, with a mean age of 20.43 years. The missing data percentage was low (<5%), and no imputation procedures were required.

The gender distribution was balanced between male (41.3%) and female participants (58.7%). Most of the subjects in the study were classified as Level 1 (62.8%), while around one third were classified as Level 2 (37.2%).

**Figure 1:**
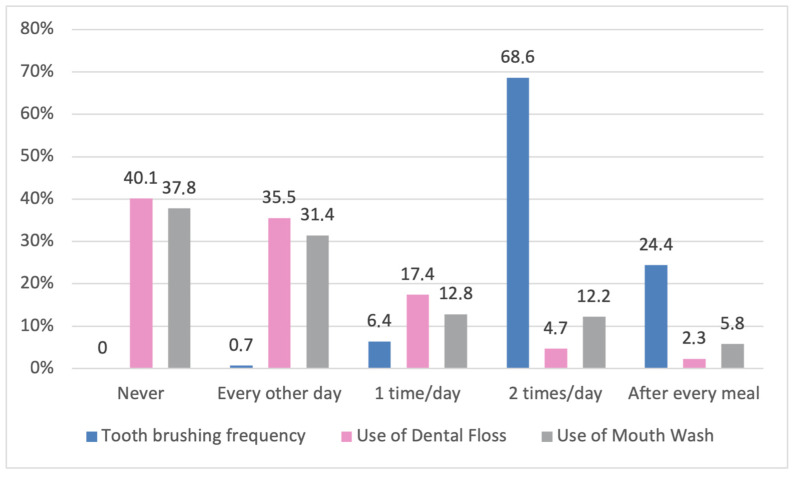
Frequency distribution of oral hygiene habits.

Participants’ oral hygiene habits varied across the sample. Regarding brushing frequency, most brush twice daily, and the remaining ¼ brush after every meal. Regarding dental floss and mouthwash, most of the participants either never use them or use them only every other day, while the remaining approximately one-third of them use them once or more than once a day (Figure 1).

**Figure 2:**
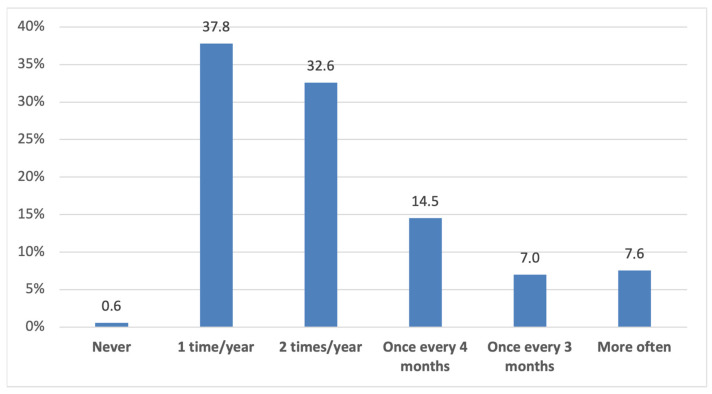
Frequency of dental office visits in the last five years.

Figure 2 shows the frequency of dental visits among the study participants over the past five years. The majority reported visiting the dental office once or twice a year. Only a very small proportion reported never attending a dental appointment, while an even smaller subgroup reported visiting every few months or more often.

The Cronbach’s Alpha reliability coefficient for the questionnaires used is shown in Table 1 and indicates good internal consistency.

The overall mean PIDAQ score was 16.56 (*n* = 172), and the mean RSES score was 32.71 (*n* = 172). In the entire sample, the Dental Self-Confidence subscale of the PIDAQ had the highest score, indicating that this aspect had the greatest perceived psychosocial impact.

**Figure 3:**
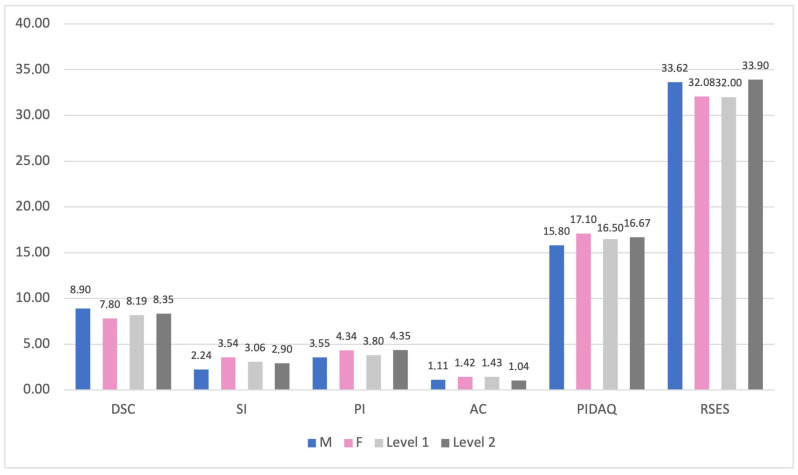
Comparison of mean scores for the PIDAQ and RSES subscales/overall scores with respect to gender and level of knowledge.

Statistical analysis using independent-samples *t*-tests revealed significant gender differences. Female participants scored significantly higher than male participants on the Social Impact subscale of the PIDAQ (t(165.16) = −2.083, *p* = 0.039), indicating a greater perceived impact of dental aesthetics. Male participants also had significantly higher overall RSES scores than female participants (t(170) = 1.998, *p* = 0.047), suggesting higher self-esteem (Table 2 and Table 3, Figure 3).

Statistically significant differences between the groups regarding dental knowledge, in relation to overall RSES scores, were also observed (t(170) = −2.429, *p* = 0.016). The Level 2 sample scored higher RSES overall values than the Level 1 sample, suggesting that early academic progression and adaptation may be associated with higher self-esteem, but cannot be interpreted as an effect of clinical exposure (Table 2 and Table 3, Figure 3).

Several small but statistically significant correlations were observed between oral hygiene habits and both aesthetic perception and dental visit frequency. Flossing frequency was positively correlated with the number of dental visits in the past five years (r = 0.316, *p* = 0.001), suggesting that proactive home care is associated with higher professional maintenance. Negative correlations were observed between the level of dental education and the frequency of dental office visits in the prior 5 years (r = −0.166, *p* = 0.030), as well as a weak negative correlation between the frequency of dental flossing and Aesthetic Concern scores on the PIDAQ (r = −0.154, *p* = 0.044). The small effect size suggests a modest association, rather than a strong relationship. A non-significant correlation was found between self-esteem (RSES) and tooth brushing frequency.

The current study found statistically significant minor Pearson correlations between overall RSES scores and the following PIDAQ subscale and overall scores: Social Impact subscale, r = −0.219, *p* = 0.004; Psychological Impact subscale, r = −0.175, *p* = 0.022, and Aesthetic Concern (r = −0.228, *p* = 0.03) subscales, as well statistical significance between the overall RSES and overall PIDAQ scores, r = −0.208, *p* = 0.006. The correlations were negative, indicating that higher self-perceived dental aesthetics problems were associated with lower self-esteem.

## 4. Discussion

Previous research has shown that dental aesthetics significantly affect an individual’s self-esteem and psychosocial well-being, underscoring the importance of examining the factors that shape social identity, particularly during key developmental stages [7,8,9].

Although the current study’s observed associations between self-perceived dental aesthetics and self-esteem were weak but statistically significant, integrating behavioural variables offers a multidimensional perspective on self-perception. The first experimental question of the current study, that self-perceived dental aesthetics (as measured by PIDAQ) is associated with general self-esteem (as measured by RSES) among dental students, was confirmed. A negative Pearson correlation indicated that a higher impact of oral health issues was associated with lower self-esteem.

The findings of the current study align with those of Botezatu et al., who concluded that dento-facial asymmetries were associated with heightened self-consciousness and reduced self-esteem regarding personal appearance [30]. Similar findings were reported in research by Faraj et al., which investigated malocclusion and self-image [31]. These findings are further supported by Grecu et al., who highlight the growing interest in the connection between oral health-related quality of life and self-esteem [32].

Research by Bahar et al. assessed the self-esteem of patients undergoing orthodontic treatment and found that those with higher self-esteem tend to view their dental appearance more positively, boosting overall life satisfaction [33]. This finding is supported by Kamath et al., who reported that patients undergoing aesthetic procedures often experience significant increases in self-esteem afterward, suggesting that aesthetic improvements can confer psychological benefits. This phenomenon is especially relevant in orthodontic treatment, where social perceptions and peer influence frequently shape the desire for improved aesthetics [34].

The second research question, assessing the presence of significant differences in self-perceived dental aesthetics, self-esteem and dental hygiene habits, with respect to the variables gender and dentistry knowledge, was partially confirmed.

Research indicates that good, regular dental hygiene practices can substantially influence an individual’s aesthetic concerns about dental and overall appearance, thereby affecting social life, health, and quality-of-life metrics [35]. This underscores the importance of education and reinforcement of good oral health habits in patients, as poor hygiene can worsen concerns about dental aesthetics and, in turn, affect social interactions and self-esteem [36,37,38].

The current research revealed small but statistically significant correlations between the Aesthetic Concern of the PIDAQ Questionnaire subscale and the frequency of dental flossing, suggesting an association between aspects of dental hygiene habits and one’s own dental aesthetics satisfaction. Our results align with existing research, emphasising that regular flossing prevents periodontal disease and reduces oral inflammation, thereby improving overall smile aesthetics and enhancing an individual’s comfort in social expression [39,40,41,42].

However, the statistical analysis did not indicate a relationship between the Rosenberg Self-Esteem Score and tooth brushing frequency. Recent literature on the relationship between oral hygiene behaviour and self-concept has shown mixed results. On the one hand, Ghorbani et al., Koga et al., and Billa et al. examined the same relationship between self-esteem and tooth brushing frequency but found no significant association [43,44,45]. On the other hand, studies by Pazos et al. and Jeong et al. demonstrated a relationship between high self-esteem and a higher frequency of tooth brushing, implying better oral health among young students [46,47]. Foláyan et al. and Khan et al. further highlight the role of effective tooth brushing in enhancing overall quality of life by reducing oral inflammation and the resulting anxiety [39,40].

In addition to tooth brushing, mouthwash has been linked to better oral health, as it helps reduce bad breath and other oral health issues that can negatively affect social life quality [48]. Furthermore, people who regularly use mouthwash report fewer aesthetic concerns on the PIDAQ survey [11,49]. In our study, over a third of participants reported never using mouthwash, approximately 31,4% used it every other day, and the remaining 30,8% used it once or more than once a day. According to Zheng et al., those who incorporate mouthwash into their daily routine tend to have higher self-esteem and greater satisfaction with their oral hygiene [10]. A sizeable proportion of dental students involved in the current study reported a rare use of dental floss or mouthwash. This is notable given their educational background and expected oral health behaviour. It may reflect cognitive dissonance in health behaviours [50].

Recent studies have shown that regular preventive dental visits play a crucial role in positively shaping self-perception of dental aesthetics [43,51,52]. The current study’s results support these findings, as individuals who have sought dental care within the past five years tend to report a more positive self-image and lower aesthetic concern scores on the PIDAQ [48].

Regarding the influence of gender on self-perceived dental aesthetics and self-esteem, statistically significant differences were found, thus confirming the second research question.

The current research showed that the Dental Self-Confidence subscale of the PIDAQ had the greatest perceived impact in this area, underscoring the importance of self-esteem in personal perception [16,17,18]. Female participants scored higher on the Social Impact subscale of the PIDAQ than males, indicating this aspect was more important to them. Additionally, scores on the Rosenberg Self-Esteem Scale were higher among males than among females, suggesting higher perceived self-worth among males. These results align with other research indicating that women are more concerned with aesthetics, experience a greater negative psychological impact, and have a notably higher total PIDAQ score [8,14,17,53,54].

The study’s rationale to focus on dental students is justified by their double role as providers and subjects of aesthetic standards. Their specialised education, which progresses from pre-clinical to clinical phases, makes them more aware of minor dental aesthetic discrepancies that the average person might ignore.

Regarding the role of dental education in self-perceived dental aesthetics and self-esteem, small statistically significant differences were found. The Rosenberg Self-Esteem Scale scores of the Level 2 sample were higher than those of the Level 1, suggesting that progressive dental medicine education may provide a more objective basis for self-assessment. The correlations presented above led to the confirmation of the second research hypothesis.

Since their self-esteem influences the impact of dental aesthetics, integrating psychosocial dimensions into dental education is essential to help future practitioners recognise the emotional impact of their treatment results on patients. Such assessment methods regarding self-image may also improve their ability to provide empathetic and holistic care to patients who present similar concerns.

Recent studies on young populations found the same association between poor self-assessed smile aesthetics and significantly lower self-esteem scores and increased social anxiety [55,56,57,58]. These findings align with those of Svalina et al. and Bous et al., which indicated that facial aesthetics significantly impact self-esteem and depressive symptoms in patients with craniosynostosis [59,60].

Regarding age, adolescents and young adults often face greater pressure related to appearance while trying to conform to specific social norms, making them more susceptible to negative self-image and diminished self-worth linked to dental aesthetics [61,62]. Furthermore, dental students have demonstrated greater awareness of aesthetic considerations than peers in other academic disciplines, suggesting that education and exposure to dental health knowledge may influence perceptions and attitudes toward aesthetics [63,64,65]. Female adolescents and young women, for instance, often experience greater psychosocial impacts than their male counterparts, as they are more likely to internalise societal expectations driven by beauty norms perpetuated by the media [65,66,67].

Beyond personal factors, broader contextual events, such as the COVID-19 pandemic, can affect self-esteem [68]. Regarding the current study’s timeframe, although specific COVID-19 stress variables were not assessed, the transition from social distancing and mandatory safety masks back to clinical and social exposure most likely influenced the participants’ high awareness of their orofacial appearance. Lockdowns, social isolation and disruption in education had negative consequences, especially on the young generations, who faced high psychological distress during their formative years [69,70].

Through these studies, researchers can deepen their understanding of how dental health affects overall well-being, thereby informing strategies to improve self-esteem and encourage positive social interactions across populations.

Based on the results of the present study, there is a significant relationship between individuals’ self-perceived dental aesthetics and self-esteem, and statistically significant differences between these constructs with respect to gender and level of knowledge in dentistry. These results support integrating psychosocial dimensions into dental education and clinical protocols, emphasising the need for a holistic approach to patient care that encompasses both the functional and emotional aspects of oral health.

Several limitations of the current study must be acknowledged. First, focusing on a single-centre convenience sample may lead to selection bias, limiting the generalizability of the results to other academic contexts with a different geographical background. Nevertheless, it serves as an exploratory research of a representative Eastern European dental medicine students cohort.

Another drawback of the current study is the reliance on self-reported questionnaires (PIDAQ and RSES) without additional clinical aesthetic assessment (e.g., IOTN index). While self-perception highly influences patient satisfaction in aesthetic dentistry, objective clinical data would have contributed to more in-depth results.

A potential non-response bias exists, as students enrolled in the Faculty of Dental Medicine may have a high interest in dental aesthetics, making them more prone to participate in the study. Future research should target more academic centres with a longitudinal study design to offer a broader perspective of this topic. However, with a sample size exceeding the power test score and a high response rate (97.7%), the current study presents a low risk of potential for non-response bias, which occurs when non-participants differ systematically from those who respond [71].

Limiting the study to students in pre-clinical years may be a constraint. We acknowledge that the observed differences between the two levels may reflect academic adaptation and maturation, increased familiarity with dental concepts, and greater exposure to theoretical aesthetic principles, rather than clinical experience. The small differences observed between years cannot be interpreted as an effect of clinical exposure. A more extensive sample, including students in clinical years, populations unrelated to dentistry, and a broader age range, is necessary for a comprehensive comparison. Regarding the level of dental education, the sample distribution was uneven, with approximately 62.8% at the first level and 37.2% at the second level. Additionally, future research should consider ethnicity as a variable, as it could enable new comparative analyses, as well as prior orthodontic treatments, socioeconomic background, or mental health status, which act as modulators for self-esteem. The effects of the COVID-19 pandemic on overall mental and social health could be addressed through additional pandemic-related psychosocial variables in future surveys.

While the relationships between self-esteem and self-perception align with the scientific literature results, the magnitude of the correlations was small, suggesting limited predictive strength.

Several methodological and statistical limitations must be acknowledged. Likert-scale questionnaire items are ordinal; although composite scores were treated as continuous in our study in accordance with common practice, alternative non-parametric approaches could also be considered [27,28]. Multiple statistical comparisons were conducted without formal correction procedures, increasing the potential risk of Type I error. Finally, no multivariate analyses were performed to adjust for potential confounding variables.

## 5. Conclusions

The current study identified weak but statistically significant associations between self-perceived dental aesthetics and self-esteem among pre-clinical dental students. Within the study’s limitations, it was concluded that higher PIDAQ scores were associated with lower RSES scores, although the strength of this association was weak. The Dental Self-Confidence subscale of the PIDAQ had the greatest perceived impact, underscoring the importance of self-image for dental students’ psychological and social well-being.

Regarding oral hygiene habits, regular dental flossing and frequent dental visits were associated with lower aesthetic concern scores, indicating that good oral hygiene behaviours may be positively correlated with self-perception. However, there was no evidence of a direct relationship between these habits and self-esteem.

Gender-related differences were observed, with female participants reporting greater social impact related to dental aesthetics and male participants reporting higher self-esteem.

Overall RSES scores were higher among participants of the Level 2 sample, having more advanced pre-clinical academic progression, and this difference may reflect academic adaptation rather than educational impact. Given the small magnitude of the observed correlations and the exploratory cross-sectional design, these findings should be interpreted cautiously and confirmed in larger, multi-centre future studies using multivariate analytical approaches.

## Figures and Tables

**Table 1:** The Cronbach’s Alpha reliability coefficient for the questionnaires used.

Questionnaire Subscale	Cronbach’s Alpha Reliability Coefficient
DSC ^1^	0.9
SI ^2^	0.83
PI ^3^	0.77
AC ^4^	0.9
PIDAQ ^5^	0.92
RSES ^6^	0.86

^1^ Dental Self-Confidence Subscale of PIDAQ Questionnaire, ^2^ Social Impact Subscale of PIDAQ Questionnaire, ^3^ Psychological Impact Subscale of PIDAQ Questionnaire, ^4^ Aesthetic Concern Subscale of PIDAQ Questionnaire, ^5^ Overall Score of the Psychosocial Impact of Dental Aesthetics Questionnaire, ^6^ Overall Score of the Rosenberg Self-Esteem Scale.

**Table 2:** Questionnaire Mean Scores, Standard Deviation, *t*-test Value, Significance and Size Effects—Gender variable.

Gender		N	Mean	Std. Deviation	Std. Error Mean	t	Sig. (2-Tailed)	Size Effect
DSC	M	71	8.90	4.76	0.56	1.375	0.171	0.21
	F	101	7.80	5.43	0.54			
SI	M	71	2.24	3.02	0.36	−2.083	0.039	−0.29
	F	101	3.54	5.16	0.51			
PI	M	71	3.55	3.56	0.42	−1.259	0.210	−0.19
	F	101	4.34	4.34.	0.43			
AC	M	71	1.11	1.83	0.22	−0.822	0.412	−0.12
	F	101	1.42	2.70	0.27			
PIDAQ	M	71	15.80	10.37	1.23	−0.668	0.505	−0.09
	F	101	17.10	15.07	1.50			
RSES	M	71	33.62	4.49	0.53	1.998	0.407	0.30
	F	101	32.08	5.30	0.53			

**Table 3:** Questionnaire Mean Scores, Standard Deviation, *t*-test Value, Significance and Size Effects—Level of dental knowledge variable.

Dental Medical Education		N	Mean	Std. Deviation	Std. Error Mean	t	Sig. (2-Tailed)	Size Effect
DSC	Level 1	108	8.19	5.02	0.48	−0.201	0.841	−0.03
	Level 2	64	8.35	5.45	0.68			
SI	Level 1	108	3.06	4.00	0.38	0.226	0.822	0.03
	Level 2	64	2.90	5.13	0.64			
PI	Level 1	108	3.80	3.52	0.33	−0.867	0.387	−0.13
	Level 2	64	4.35	4.80	0.60			
AC	Level 1	108	1.43	2.26	0.21	1.035	0.302	0.16
	Level 2	64	1.04	2.55	0.31			
PIDAQ	Level 1	108	16.50	12.04	1.15	−0.082	0.935	−0.01
	Level 2	64	16.67	15.31	1.91			
RSES	Level 1	108	32.00	4.74	0.45	−2.429	0.16	−0.37
	Level 2	64	33.90	5.28	0.66			

## Data Availability

The raw data supporting the conclusions of this article will be made available by the authors on request.

## Supplementary Materials

The following supporting information can be downloaded at https://www.mdpi.com/article/10.3390/dj14030165/s1, Questionnaire S1: Assessing The Relationship between the Psychosocial Impact of Dental Aesthetics, Self-Esteem, and Dental Habits.

## Abbreviations

The following abbreviations are used in this manuscript:

PIDAQ	Psychosocial Impact of Dental Aesthetics Questionnaire
RSES	Rosenberg Self-Esteem Scale
DSC	Dental Self-Confidence
SI	Social Impact
PI	Psychological Impact
AC	Aesthetic Concern
M	Masculine; Male
F	Feminine; Female
