# Supplementary material for: Assessing the Relationship Between the Psychosocial Impact of Dental Aesthetics, Self-Esteem, and Dental Habits
Source: Dent J (Basel). 2026 Mar 12;14(3):165. doi: 10.3390/dj14030165 (PMC13025585; doi:10.3390/dj14030165)
Supplement: Supplementary file 1 [file dentistry-14-00165-s001.zip › dentistry-4161036-supplementary/dentistry-4161036-supplementary.pdf]

SUPPLEMENTARY FILE: QUESTIONNAIRE S1.

ASSESSING THE RELATIONSHIP BETWEEN THE PSYCHOSOCIAL IMPACT OF DENTAL AESTHETICS, SELF-ESTEEM, AND DENTAL HABITS

Please complete the following information from the questionnaire and circle the preferred answer in case of multiple-choice questions.

1. PERSONAL DATA:

- NAME: ____

- SURNAME: ____

- AGE: ____

- SEX: M / F / Rather not specify

2. DEMOGRAPHIC/ COMPORTAMENTAL DATA:

- LIVING ENVIRONMENT:

a. Urban
b. Rural

- EDUCATION: YEAR OF STUDIES:

a. 1
b. 2
c. 3
d. 4
e. 5
f. 6

- EDUCATION: DOMAIN OF STUDIES:

a. Dental Medicine

b. Medicine

c. Dental Technology

d. Other – please specify: ....

- HOW OFTEN HAVE YOU BEEN TO YOUR DENTIST IN THE LAST 5 YEARS?

a. Never

b. 1 time / year

c. 2 times / year

d. Once every 4 months

e. Once every 3 months

f. More often than the other variants

- HOW OFTEN DO YOU BRUSH YOUR TEETH?

a. Never

b. Once every few days

c. 1 time/ day

d. 2 times/ day

e. After every meal

- HOW OFTEN DO YOU FLOSS?

a. Never

b. Once every few days

c. 1 time/ day

d. 2 times/ day

e. After every meal

- HOW OFTEN DO YOU USE MOUTHWASH?

a. Never

b. Once every few days

c. 1 time/ day

d. 2 times/ day

e. After every meal

3. PSYCHOSOCIAL IMPACT OF DENTAL AESTHETICS QUESTIONNAIRE (PIDAQ)

Please circle for each sentence a value between 0 (not at all) and 4 (very strongly) regarding how much you personally agree with each sentence.

a.	***Dental Self-Confidence***	**0**	**1**	**2**	**3**	**4**
1.	I am proud of my teeth.	Not at all	A little	Somewhat	Strongly	Very Strongly
2.	I like to show my teeth when I smile.	Not at all	A little	Somewhat	Strongly	Very Strongly
3.	I am pleased when I see my teeth in the mirror.	Not at all	A little	Somewhat	Strongly	Very Strongly
4.	My teeth are attractive to others.	Not at all	A little	Somewhat	Strongly	Very Strongly
5.	I am satisfied with the appearance of my teeth.	Not at all	A little	Somewhat	Strongly	Very Strongly
6.	I find my tooth position to be very nice.	Not at all	A little	Somewhat	Strongly	Very Strongly
**b.**	***Social Impact***					
7.	I hold myself back when I smile so my teeth don't show so much.	Not at all	A little	Somewhat	Strongly	Very Strongly
8.	If I don't know people well I am sometimes concerned what they might think about my teeth.	Not at all	A little	Somewhat	Strongly	Very Strongly
9.	I'm afraid other people could make offensive remarks about my teeth.	Not at all	A little	Somewhat	Strongly	Very Strongly
10.	I am somewhat inhibited in social contacts because of my teeth.	Not at all	A little	Somewhat	Strongly	Very Strongly
11.	I sometimes catch myself holding my hand in front of my mouth to hide my teeth.	Not at all	A little	Somewhat	Strongly	Very Strongly
12.	Sometimes I think people are staring at my teeth.	Not at all	A little	Somewhat	Strongly	Very Strongly
13.	Remarks about my teeth irritate me even when they are meant jokingly.	Not at all	A little	Somewhat	Strongly	Very Strongly
14.	I sometimes worry about what members of the opposite sex think about my teeth.	Not at all	A little	Somewhat	Strongly	Very Strongly
**c.**	***Psychological Impact***					
15.	I envy the nice teeth of other people.	Not at all	A little	Somewhat	Strongly	Very Strongly
16.	I am somewhat distressed when I see other people's teeth.	Not at all	A little	Somewhat	Strongly	Very Strongly
17.	Sometimes I am somewhat unhappy about the appearance of my teeth.	Not at all	A little	Somewhat	Strongly	Very Strongly
18.	I think most people I know have nicer teeth than I do.	Not at all	A little	Somewhat	Strongly	Very Strongly

19.	I feel bad when I think about what my teeth look like.	Not at all	A little	Somewhat	Strongly	Very Strongly
20.	I wish my teeth looked better.	Not at all	A little	Somewhat	Strongly	Very Strongly
**d.**	***Aesthetic Concern***					
21.	I don't like to see my teeth in the mirror.	Not at all	A little	Somewhat	Strongly	Very Strongly
22.	I don't like to see my teeth in photographs.	Not at all	A little	Somewhat	Strongly	Very Strongly
23.	I don't like to see my teeth when I look at a video of myself.	Not at all	A little	Somewhat	Strongly	Very Strongly

4. ROSENBERG SELF-ESTEEM SCALE (RSES)

Please circle for each sentence the answer regarding how much you personally agree with each sentence.

	**Strongly agree**	**Agree**	**Disagree**	**Strongly disagree**
1. On the whole, I am satisfied with myself.	Strongly agree	Agree	Disagree	Strongly disagree
2.* At times I think I am no good at all.	Strongly agree	Agree	Disagree	Strongly disagree
3. I feel that I have a number of good qualities.	Strongly agree	Agree	Disagree	Strongly disagree
4. I am able to do things as well as most other people.	Strongly agree	Agree	Disagree	Strongly disagree
5.* I feel I do not have much to be proud of.	Strongly agree	Agree	Disagree	Strongly disagree
6.* I certainly feel useless at times	Strongly agree	Agree	Disagree	Strongly disagree

7. I feel that I'm a person of worth, at least on an equal plane with others.	Strongly agree	Agree	Disagree	Strongly disagree
8.* I wish I could have more respect for myself.	Strongly agree	Agree	Disagree	Strongly disagree
9.* All in all, I am inclined to feel that I am a failure.	Strongly agree	Agree	Disagree	Strongly disagree
10. I take a positive attitude toward myself	Strongly agree	Agree	Disagree	Strongly disagree

APPENDIX 3

Regulation regarding the gathering, processing and storage of personal data and documents, according to art. 6 of Regulation (EU) 2016/679

The undersigned____, as a volunteer for the study named "**Assessing the Relationship Between the Psychosocial Impact of Dental Aesthetics, Self-Esteem, and Dental Habits**" led by the main researcher Șimon Mara-Ștefania, carried out within the Discipline of Dental Aesthetics and Propedeutics, UMF "Iuliu Haţieganu", Cluj-Napoca, Romania, by this agreement I expressly express my consent to participate in the study and for the principal investigator together with the research team to collect, process and store my personal data and documents, consisting of: name, surname, signiture, personal socio-demographic data in the questionnaires, information filled in in the questionnaires, until the end of the respective studies and the archiving period according to national legislation. This information is provided by me freely and unequivocally for the purpose of conducting PhD research studies.

Name and surname:

Date:

Signiture:

INFORMED CONSENT FORM

Main Researcher: Șimon Mara Ștefania

Organisation: Faculty of Dental Medicine, Department of Prosthetic Dentistry and Materials, Discipline of Dental Propedeutics and Asthetics, “Iuliu Hațieganu” University of Medicine and Pharmacy, Cluj-Napoca, Romania

Study Title: Assessing the Relationship Between the Psychosocial Impact of Dental Aesthetics, Self-Esteem, and Dental Habits

Please complete all the following blank spaces with the proper information by checking the proper boxes or completing missing personal information:

☐ I am a participant in this study

NAME of the participant______________________________

Are you currently participating in other clinical or research studies? ☐ Yes ☐ No

PURPOSE OF THE STUDY

You are invited to participate in a scientific research study conducted in accordance with the ethical standards, including the Declaration of Helsinki.

The purpose of this research is to investigate the perception of dento-facial esthetic parameters in relation to individual socio-demographic and psychological factors. The study aims to identify correlations between personal characteristics and individual preferences regarding dental and facial esthetics.

You have been invited to participate because you meet the inclusion criteria, namely adults over 18 years of age enrolled in university studies in Cluj-Napoca, Romania.

VOLUNTARY PARTICIPATION AND RIGHT TO WITHDRAW

Your participation in this study is entirely voluntary. You may refuse to participate or withdraw your consent at any time without providing a reason and without any negative consequences.
Your decision will not affect your access to medical care, academic status, or any other entitled benefits.

If you do decide to withdar, please notify the main researcher.

PROCEDURES

If you agree to participate, you will be asked to:

- Provide socio-demographic data through a standardized form.
- Complete questionnaires regarding self-perceived oral health and dental esthetics.
- Complete validated psychological and temperamental assessment questionnaires.
- All collected data will be anonymized and statistically analyzed.

STUDY DURATION

Participation involves a single session lasting approximately 30-45 minutes. The overall study duration is 12 months.

RISKS AND DISCOMFORTS

This study involves no foreseeable physical or psychological risks. Minor inconvenience may include time commitment and travel to the study location.

BENEFITS

There are no direct medical benefits for participants, except for improved understanding of psycho-social behaviour of the participants sample based on the study results. The results may contribute to improved understanding of patient-centered esthetic treatment planning in restorative dentistry.

RESPONSABILITIES OF THE PARTICIPANT

As a participant, your responsibilities include:

- Following the instructions and recommendations provided by the research team.
- Completing study questionnaires as instructed.
- Attending study visits as scheduled. If you are unable to attend, please notify Șimon Mara-Ștefania.
- Asking questions whenever necessary.

PARTICIPANTS' RIGHTS

- You are not required to participate in this study and should take sufficient time to decide.
- Your questions must receive clear and satisfactory answers.
- You may withdraw from the study at any time by informing the investigator.
- Any new information that may affect your health or willingness to continue participation will be communicated to you in a timely manner.

WITHDRAWAL FROM THE STUDY

If you initially agree to participate and later change your mind, you are free to withdraw your consent and discontinue participation at any time. This decision will not affect your right to continue your university studies or result in loss of any entitled benefits.

The investigator may also withdraw you from the study without your consent for reasons including non-compliance, potential harm, study termination, administrative reasons, unforeseen circumstances, or withdrawal of study funding.

CONFIDENTIALITY AND DATA PROTECTION

The results of this study may be published in peer-reviewed scientific journals, or presented at scientific conferences. No

information that could identify you will be published.

All data collected will be treated as confidential and processed in anonymized form. Personal identifiers will not be disclosed in any publications. Data may be reviewed by the research team, ethics committee members, or regulatory authorities in accordance with applicable laws.

FINANCIAL CONSIDERATIONS

Participation is unpaid. No additional costs are associated with participation other than personal time and transportation.

ETHICAL CONSIDERATIONS

The study was conducted in accordance with the Declaration of Helsinki, and approved by the Research Ethics Committee of "Iuliu Hațieganu" University of Medicine and Pharmacy in Cluj-Napoca (protocol code nr. DEP245/30.10.2024 from date of 30 October 2024).

FINANCING OF THE STUDY

This study was funded by the Research Project PCD Nr. 284/51/12.01.2026 from "Iuliu Hațieganu" University of Medicine and Pharmacy in Cluj-Napoca. This article was also supported by project no. 100418/29.08.2025, SMIS code 350525, financed by the Ministry of Investments and European Projects, through the Health Programme. The research used the infrastructure provided by the University of Medicine and Pharmacy "Iuliu Hatieganu"-Cluj-Napoca, Romania.

CONTACT INFORMATION

For questions or concerns regarding this study, you may contact: Principal Investigator: Mara-Ștefania Șimon

STATEMENT OF CONSENT

By signing below, you confirm that:
• You have read and understood this information.
• You have had the opportunity to ask questions and received satisfactory answers.
• You voluntarily agree to participate in this study.

You will receive a signed and dated copy of this informed consent form.

Participant Signature ______________________________

Participant Name ______________________________

Date ____________________
